# Author Correction: Mechanistic insights into bacterial metabolic reprogramming from omics-integrated genome-scale models

**DOI:** 10.1038/s41540-020-0123-2

**Published:** 2020-01-30

**Authors:** Noushin Hadadi, Vikash Pandey, Anush Chiappino-Pepe, Marian Morales, Hector Gallart-Ayala, Florence Mehl, Julijana Ivanisevic, Vladimir Sentchilo, Jan R. van der Meer

**Affiliations:** 1 Department of Fundamental Microbiology, University of Lausanne, 1015 Lausanne, Switzerland; 2 Laboratory of Computational Systems Biotechnology, Ecole Polytechnique Fédérale de Lausanne (EPFL), 1015 Lausanne, Switzerland; 3 Metabolomics Platform, University of Lausanne, 1015 Lausanne, Switzerland

**Keywords:** Environmental sciences, Systems analysis

**Correction to:** *npj Systems Biology and Applications* 10.1038/s41540-019-0121-4, published online 7 Jan 2020

In Fig. 3 of the original version of this Article, unrelated text (Lorem ipsum) was accidentally included. This text has now been removed.

