# Supplementary material for: Mechanistic insights into bacterial metabolic reprogramming from omics-integrated genome-scale models
Source: NPJ Syst Biol Appl. 2020 Jan 7;6:1. doi: 10.1038/s41540-019-0121-4 (PMC6946695; doi:10.1038/s41540-019-0121-4)
Supplement: Supplementary file 1 — SupplementaryInformation [file 41540_2019_121_MOESM1_ESM.docx]

**Supplementary Figure 1:** **Principal component analysis of** (A) quadruplicate global RNA-sequencing data sets of P. veronii 1YdBTEX2 after 4 h, 24 h and the 0 h control (T0) and (B) of exometabolomics data, grouped QC samples of T0, T4 and T24 samples.

**Supplementary Figure 2: Main functional toluene degradation pathway branch in iPsvr.** 3-methylcatechol is converted to pyruvate and acetaldehyde, which are further involved in central carbon pathways.

**Supplementary Figure 3: Differential in silico production (mmol/gDW/h) of seven biomass building block groups between cells in liquid medium and sand.** (A) Integration of relative gene expression data (liquid vs sand) using REMI-TGex when cells grow on Toluene . (B) Integration of relative gene expression data (liquid vs sand) using REMI-TGex when cells grow on Succinate. Each dot in the graph represents the individual biomass precursors within that category and the statistically significant changes (p=value < 0.05) are highlighted in purple (A) and in orange (B). For BBB group statistics, see Table S10.

**SUPPLEMENTARY TABLES** (given as an excel file and each excel sheet is a Supplementary Table)

**Table S1.** Summary of RNA-seq yields of the different time points.

**Table S2.** Significantly differentially expressed metabolic genes in EXPO vs. STAT

**Table S3**. Under-represented Biological processes in STAT vs EXPO.

**Table S4**. Biolog data and the result of using them for the curation/validation of the model.

**Table S5.** iPsvr network decomposition into its main subnetwork and isolated reactions/pathways.

**Table S6**. Gap-filled reactions introduced into the iPsvr with their corresponding genes.

**Table S7.** Common and uncommon reactions amongst alternative solutions generated by REMI for exponential to stationary transition and KEGG pathway analysis of common reactions.

**Table S8**. Differential *in silico* production (mmol/gDW/h) of biomass precursors, grouped in seven biomass building block groups, for exponential to stationary transition, using REMI-TGexM, together with statistical test.

**Table S9.** Common and uncommon reactions amongst alternative solutions generated by REMI for liquid to sand transition (Toluene and Succinate as carbon sources) and KEGG pathway analysis of common reactions

**Table S10.** Differential *in silico* production (mmol/gDW/h) of biomass precursors, grouped in seven biomass building block groups, for liquid to sand transition (Toluene and Succinate as carbon sources), using REMI-TGex, together with statistical test.

**Table S11.** Detailed analysis of reaction balancing in the iPsvr, together with explanation for initially imbalanced reactions.
